# Regulation of Intestinal P-Glycoprotein by Gamma-Ray Irradiation: Impacts on Transporter Expression and Oxidative Stress

**DOI:** 10.3390/ijms27198586

**Published:** 2026-09-25

**Authors:** Haiyang Wu, Jing Yang, Runtian Li, Wenhua Zhu, Zhixuan Yuan, Min Wang, Yan Tong, Zhiyun Meng, Ruolan Gu, Yunbo Sun, Guifang Dou, Hui Gan

**Affiliations:** 1 School of Public Health, University of South China, Hengyang 421001, China; ldsxwhy@126.com (H.W.); guruolan@bmi.ac.cn (R.G.); 2 Academy of Military Medical Sciences, Beijing 100850, China; yangjing1030a@163.com (J.Y.); lrt879228@163.com (R.L.); zhuwh0114@163.com (W.Z.); 17671292373@163.com (Z.Y.); 18763893250@163.com (M.W.); 19904142464@163.com (Y.T.); mengzhiyun@vip.163.com (Z.M.); bobo09192024@163.com (Y.S.); dougf@bmi.ac.cn (G.D.); 3 School of Life Sciences, Hebei University, Baoding 071002, China

**Keywords:** gamma rays, P-gp, transport activity, radiation damage

## Abstract

P-glycoprotein (P-gp), a key efflux drug transporter which plays a pivotal role in the in vivo pharmacokinetics of medications, undergoes alterations in expression and function following radiation exposure that can impact drug metabolism and therapeutic efficacy. Current research has found that clinical doses of radiation can affect the expression of drug transporters, while the underlying mechanisms associated with single high-dose irradiation remain to be thoroughly elucidated. This study investigates the effects of gamma-ray irradiation on the expression and transport activity of P-glycoprotein (P-gp) in various cell lines and murine small-intestinal tissues, and preliminarily explores the underlying regulatory mechanisms, thereby providing an experimental basis for research into drug transport abnormalities and interventions associated with radiation injury. Four different cell lines and male C57BL/6J mice were exposed to γ-ray irradiation from a ^60^Co source. P-gp gene expression was quantitatively analyzed, combined with the relative expression levels of proteins in intestinal cells and small-intestinal tissues and relevant physiological parameters. Furthermore, a Transwell intestinal epithelial cell barrier model and an in vivo mouse intestinal perfusion model were established. Using digoxin as a P-gp specific substrate, a quantitative LC–MS method was developed to assess P-gp transport activity after irradiation. Finally, transcriptome sequencing analysis of mouse small-intestinal tissue was performed, and oxidative-stress-related pathways were preliminarily detected. At the cellular level, the mRNA expression was significantly upregulated, peaking at 4.19 times that of the control group, while protein expression was also significantly upregulated, reaching a peak of approximately 2.50 times that of the control; at the animal level, the peak upregulation for mRNA and protein was 2.09-fold and 1.95-fold, respectively. Transport activity also gradually increased over time after irradiation. At the animal level, transcriptome analysis further revealed that changes in pathways such as oxidative stress and immune inflammatory responses may be jointly related to radiation-induced intestinal P-gp regulation mechanisms. Gamma-ray irradiation was associated with significant upregulation of P-gp expression and was consistent with enhanced efflux activity suggestive of P-gp involvement in various cell lines and mouse small-intestinal tissues. Radiation exposure induced intestinal damage and oxidative stress, and was associated with activation of the Nrf2/HO-1 antioxidant signaling pathway.

## 1. Introduction

Drug transporters constitute a diverse family of membrane-bound proteins expressed on the plasma membranes of cells in key pharmacokinetic tissues—including the intestine, liver, kidney, and blood–brain barrier [1]. They govern the absorption, distribution, metabolism, and excretion (ADME) of endogenous compounds and xenobiotics, thereby serving as pivotal determinants of drug disposition, bioavailability, efficacy, and toxicity [2]. As integral mediators of transmembrane substrate flux, these proteins exhibit high specificity and affinity for drugs and other physiologically relevant molecules [3,4]. Over the past decade, drug transporters have emerged as a major research focus, driven by pressing clinical imperatives—including optimizing therapeutic regimens, overcoming multidrug resistance in oncology and infectious diseases, and mitigating adverse drug reactions [5,6]. P-glycoprotein (P-gp; encoded by the *ABCB1* gene), the prototypical ATP-binding cassette (ABC) efflux transporter, plays a central role in limiting oral bioavailability and restricting drug penetration into sanctuary sites as one of the most well-characterized membrane drug transporters widely expressed in pharmacokinetically vital tissues [7]. Therefore, elucidating post-irradiation changes in drug transporter expression, localization, and functional activity—and deciphering their underlying molecular mechanisms—is of paramount translational significance.

According to the electromagnetic spectrum, radiation is broadly classified into ionizing radiation—including gamma rays, X-rays, and charged subatomic particles (e.g., alpha and beta particles) emitted during radioactive decay—and non-ionizing radiation, such as ultraviolet (UV) radiation and microwaves [8,9]. With the progressive advancement and large-scale application of nuclear technology, anthropogenic environmental radiation sources have expanded substantially, and radiation protection for healthcare personnel, nuclear industry workers, and first responders has emerged as a critical public health priority [10]. Emerging evidence from recent regional conflicts indicates that injuries induced by novel radiological or radiation-emitting weapons present unprecedented clinical challenges, necessitating urgent advancements in military and civilian medical countermeasures [11,12,13].

Acute high-dose ionizing radiation exposure triggers multisystem organic impairment, manifesting as acute radiation syndrome (ARS)—a life-threatening clinical condition characterized by hematopoietic failure, gastrointestinal dysfunction, neurovascular impairment, and cutaneous damage, with high morbidity and mortality depending on dose and exposure geometry [14,15]. The severity of damage after exposure to ionizing radiation is not constant but closely related to three core factors: radiation dose, exposure time, and exposure mode. These factors collectively influence the type of damage, the rate of development, and the prognosis [16]. The development of effective radioprotective and radiation-mitigating agents is essential for safeguarding patients undergoing radiotherapy, nuclear facility personnel, and populations affected by radiological emergencies.

However, radiation-induced alterations in hepatic and intestinal metabolic capacity—and particularly in transporter-mediated processes—may profoundly compromise the pharmacokinetics of such countermeasures: accelerated clearance may reduce therapeutic exposure, whereas transporter inhibition or downregulation may elevate systemic drug concentrations, increasing the risk of off-target toxicity [17,18,19]. Given its broad substrate specificity and regulatory responsiveness to cellular stress, P-gp represents a compelling candidate for mediating radiation-altered drug distribution and metabolism [20]. To date, most studies investigating radiation-induced modulation of transporter expression and function have centered on therapeutic contexts, employing clinically relevant fractionated or low-dose-rate exposures to X- or gamma-rays—mimicking conventional radiotherapy protocols [21]. In contrast, relevant research focusing on single high-dose acute gamma irradiation and its regulatory effects on P-gp in normal tissues remains largely insufficient. Mechanistic investigation of P-gp regulation following high-dose irradiation may thus inform evidence-based strategies for dose individualization and rational drug selection in radiation-exposed individuals. In tumor cells, P-gp is abnormally highly expressed, actively effluxing chemotherapeutic drugs that have entered tumor cells, reducing the concentration of these drugs within the cells and thus decreasing their bioavailability [22,23]. This results in the inability to effectively kill tumor cells, leading to drug resistance in tumors, which is one of the main reasons for chemotherapy failure in clinical tumors. Furthermore, recent studies have found that P-gp may also participate in physiological and pathological processes such as immune regulation and inflammatory responses; the diversity of its functions is still under further investigation [24,25].

Against this background, the present study selected multiple human and rodent cell lines and C57BL/6J mice as research objects to explore the effects of gradient doses of acute gamma-ray irradiation on *ABCB1* mRNA abundance, P-gp protein expression, and efflux transport activity. Combining in vitro Transwell cell barrier models and in vivo intestinal perfusion assays, we further examined radiation-induced changes in P-gp-mediated drug transport function. Moreover, transcriptome sequencing and biochemical detection were performed to preliminarily reveal the potential signaling pathways involved in this regulatory process. This work aims to fill the research gap of P-gp regulation under acute high-dose radiation, and provide experimental evidence for individualized medication and radiation protection in populations exposed to acute ionizing radiation.

## 2. Results

### 2.1. Effects of Gamma-Ray Irradiation on ABCB1 Gene Expression in Caco-2/IEC-6/HK-2/Beas-2b Cells

To investigate whether the regulatory effect of gamma irradiation on P-gp expression represents a generalized stress response or is cell-type-specific, we selected four cell lines encompassing diverse tissue origins and species: Caco-2 (human colorectal adenocarcinoma) and IEC-6 (rat intestinal epithelial) cells represent the intestinal epithelium—the primary site of oral drug absorption and P-gp-mediated efflux; HK-2 (human renal proximal tubular) cells represent the kidney, a major organ for drug excretion; and Beas-2b (human bronchial epithelial) cells represent the respiratory tract, relevant for inhalation exposure scenarios. These cells were exposed to gradient gamma-ray doses (5, 15, and 30 Gy), and *ABCB1* mRNA levels were measured at 6–48 h post-irradiation (Figure 1). Our results demonstrated that gamma irradiation significantly upregulated *ABCB1* mRNA abundance in all four cell lines, albeit with distinct temporal response characteristics, suggesting a universal yet kinetically heterogeneous regulatory effect across different cell types and species.

Caco-2 and Beas-2b cells exhibited an early peak response: *ABCB1* abundance in Beas-2b cells increased to 3.6-fold of the control at 6 h after 30 Gy irradiation (*p* < 0.001) and remained significantly elevated until 48 h. IEC-6 intestinal epithelial cells showed sustained high-level upregulation, with *ABCB1* mRNA level peaking at 3.2-fold (15 Gy) and 3.0-fold (30 Gy) at 12 h (*p* < 0.001) and maintaining high expression for 48 h. In contrast, HK-2 renal epithelial cells displayed a delayed response, with significant elevation of *ABCB1* mRNA observed at 48 h post-irradiation (4.2-fold for 30 Gy, *p* < 0.001). These results confirm that gamma-ray irradiation leads to elevated *ABCB1* mRNA abundance in multiple tissue-derived cell lines, with cell-type-specific temporal differences. These findings suggest that irradiation may enhance *ABCB1* transcriptional activity, which may have implications for P-gp-mediated transport.

### 2.2. Effects of Gamma-Ray Irradiation on P-gp Protein Expression in Caco-2/IEC-6 Cells

The gut is indeed one of the core organs for drug metabolism and transport [26,27]. Consistent with the dose-dependent upregulation of *ABCB1* mRNA, P-gp protein expression was also significantly enhanced in irradiated Caco-2 and IEC-6 cells, as validated by Western blotting (normalized to β-actin) and immunofluorescence staining.

In Caco-2 cells (Figure 2A), P-gp levels increased progressively after radiation exposure. Significant upregulation was first detected at 6 h in the 30 Gy group (1.4-fold vs. control, *p* < 0.05), with induction persisting through 48 h. Peak expression occurred at 24 h, where both 15 Gy and 30 Gy groups showed ~1.4-fold elevations (*p* < 0.01). IEC-6 cells exhibited a more robust, sustained P-gp response (Figure 2B). Significant upregulation was observed as early as 6 h (1.7-fold in the 30 Gy group, *p* < 0.05), intensified to 2.0-fold at 24 h (*p* < 0.001), and peaked at 2.5-fold at 48 h (*p* < 0.05). These findings were corroborated by immunofluorescence (Figure 2C), which showed a dose-dependent increase in P-gp (green) fluorescence intensity in IEC-6 cells, with the 30 Gy group displaying significantly higher signal than controls (*p* < 0.01).

Collectively, these data confirm concordant transcriptional and translational upregulation of P-gp in irradiated intestinal epithelial cells, with IEC-6 cells demonstrating stronger and more prolonged induction. These results support the hypothesis that radiation exposure may alter P-gp-mediated drug transport function.

### 2.3. Effects of Gamma-Ray Irradiation on Mouse Phenotypes and Expression of Abcb1a Gene and P-gp Protein in Small-Intestinal Tissue

A dose of 6 Gy of gamma rays is a sublethal dose for mice [28]. After exposure to irradiation, the body weight of each C57 mouse was measured, and an immediate decrease in body weight was observed in the irradiated group (Figure S1A) . The thymus and spleen, key immune organs, also showed significant decreases after irradiation [29] (Figure S1B,C). Abdominal irradiation affected colon length, with a significant decrease in length in the irradiated group compared to the control group (Figure 3A).

Small-intestinal tissue was collected, and total mRNA and total protein were extracted. RT-qPCR and Western blotting were used to detect relative expression changes. At the mRNA level, the relative expression level of *Abcb1a* gene in the small-intestinal tissue of the 1-day-post-6 Gy-gamma-ray-irradiation group was 1.97 times that of the control group, and the relative expression level of P-gp gene in the small-intestinal tissue of the 3-day-post-irradiation group was 2.09 times that of the control group (Figure 3B). At the protein level, the relative expression level of P-gp protein in the small-intestinal tissue of the 3-day-post-irradiation group was significantly increased, reaching 1.95 times that of the control group (Figure 3C).

Immunohistochemical results showed that radiation induced persistent P-gp overexpression in intestinal tissues, accompanied by decreased expression of intestinal barrier proteins ZO-1 and occludin, indicating that the barrier function of the small-intestinal tissue of mice was damaged (Figure 3D).

### 2.4. Establishment of a Transport Model and the Effects of Gamma-Ray Irradiation on P-gp Transport Activity in Mice

The Caco-2 cell model is a commonly used method for studying drug transport, which can simulate the absorption, transport, and metabolism of drugs in the small-intestinal mucosa [30]. The membrane integrity of cell culture membranes was monitored by TEER measurement from the beginning to the end of the cell culture. The resistance tended to stabilize 10 days after cell inoculation, reaching the membrane formation standard (>500 Ω), and the integrity and compactness of the model were successfully verified (Figure 4A). To evaluate the effects of irradiation on barrier integrity, TEER was measured beforehand and at 24 h post-irradiation. TEER showed a mild but non-significant decrease from 964.59 Ω·cm^2^ before irradiation to 901.84 Ω·cm^2^ at 24 h after 15 Gy irradiation (*p* > 0.05, two-way ANOVA with Tukey’s post hoc test, n = 6), remaining above 900 Ω·cm^2^ at all time points and indicating that the monolayer barrier remained largely intact during the 24 h experimental window. Fluorescent yellow permeability assay revealed a modest but statistically significant 1.3-fold increase in relative fluorescence intensity in the irradiated group compared to the control group (*p* < 0.05, unpaired *t*-test, n = 3) [31]. This indicates that irradiation induced a measurable yet limited increase in paracellular permeability. Collectively, these barrier integrity assessments demonstrate that while irradiation caused a slight perturbation of the epithelial monolayer, the barrier remained sufficiently intact to support valid transporter functional studies, and the enhanced digoxin efflux observed cannot be simply attributed to general barrier breakdown. Digoxin is a classic P-gp substrate drug and can be used as a tool to detect changes in P-gp activity through pharmacokinetic methods [32]. Digoxin standard curve in Transwell intestinal epithelial barrier model is presented in Figure S2. After the Transwell chamber received 15 Gy γ-ray irradiation and was incubated for 24 h, digoxin solution was introduced into the apical/basolateral compartment of the Transwell insert. Experimental results of bidirectional transmembrane transport show that the digoxin efflux rate at 30, 60, and 90 min was significantly higher than that in the control group, with an efflux rate > 2, showing a clear efflux trend (Figure 4C). The increasing efflux ratio over time reflects the progressive accumulation of digoxin on the basolateral side and its active efflux back to the apical side. As the concentration gradient builds, the P-gp-mediated transport becomes more prominent relative to passive diffusion. This pattern is consistent with the kinetic behavior of P-gp substrates in Transwell systems, where efflux ratio increases with extended incubation time as the system approaches steady state.

Total bile acids (TBA) are core markers of the enterohepatic circulation and serve as important efflux substrates for intestinal P-glycoprotein (P-gp). The expression and activity of P-gp are associated with the efficiency of intestinal bile acid efflux, thereby influencing serum bile acid level [33,34]. Thus, by measuring changes in serum TBA, one can investigate the impact of P-gp on systemic biochemical metabolic functions. In our study, a time-dependent increase in TBA was observed, with the level in the IR-3d group being 2.29-fold higher than that in the control group (Figure 4D). This result is likely attributable to the indirect effects of radiation-induced intestinal barrier injury and alterations in the intestinal microenvironment, which together compromise P-gp-mediated transport (Figure 4D).

In vivo intestinal perfusion experiments further verified functional changes in P-gp [35]. Digoxin standard curve of mouse in vivo intestinal perfusion samples is presented in Figure S3. Compared with the blank control group, the effective intestinal permeability (P_eff_) of the irradiated mice decreased significantly in a time-dependent manner. The mean P_eff_ of the blank control group was 0.029 cm/min, representing the intestinal absorption level of digoxin corresponding to the basal activity of P-gp under normal physiological conditions. In the irradiated group 1 day after radiation (IR-1d), P_eff_ decreased to 0.019 cm/min, which was significantly lower than that of the control group (*p* < 0.01). In the irradiated group 3 days after radiation (IR-3d), P_eff_ further decreased to 0.014 cm/min, a more significant decrease (Figure 4E).

The above in vitro and in vivo functional results corroborate the previous trend of upregulated expression of *ABCB1/Abcb1a* gene and P-gp protein in cells and in vivo [36,37,38,39]. Although digoxin is not an absolutely P-gp-specific substrate, P-gp is the principal efflux transporter mediating its transepithelial outward transport in intestinal models. The concordant trends observed across mRNA, protein, and functional assays on P-gp in our study strengthenfurther support the impact of radiation on P-gp regulation.. Nevertheless, we acknowledge that future studies incorporating a blocking experiment with a selective P-gp inhibitor would further strengthen the causal interpretation of these findings.

### 2.5. Mouse Small-Intestine Transcriptome Sequencing

To systematically dissect the global molecular landscape underlying radiation-induced intestinal injury and the observed upregulation of P-gp expression, we performed high-throughput transcriptome sequencing on small-intestinal tissues collected from mice at 1 and 3 days following exposure to 6 Gy gamma-irradiation.

First, quality control analyses including principal component analysis (PCA, Figure 5A) and inter-sample correlation heatmaps (Figure S4) were conducted to validate data reliability and sample homogeneity. The PCA plot demonstrated clear separation between the control (Blank) and irradiated groups (IR1D, IR3D), with biological replicates clustering tightly within each group, confirming robust experimental reproducibility and minimal batch effects. Consistently, the correlation heatmap revealed high intra-group correlation coefficients (R^2^ > 0.9) and distinct inter-group clustering, verifying the integrity of the transcriptomic data.

Differential expression analysis identified a total of 418 differentially expressed genes (DEGs) at 1 day post-irradiation (IR1D vs. Blank), comprising 269 upregulated and 149 downregulated transcripts. At 3 days post-irradiation (IR3D vs. Blank), the number of DEGs increased substantially to 1170, with 166 upregulated and 1004 downregulated genes (Figure 5B). The overall expression patterns of differentially expressed genes among groups are shown as a clustering heatmap in Figure S5. Volcano plots (Figure 5C,D) further visualized the distribution of these DEGs, highlighting key significantly altered transcripts associated with stress responses and inflammation. Functional enrichment analyses, including Gene Ontology (GO, Figure 5E,F) and Kyoto Encyclopedia of Genes and Genomes (KEGG, Figure 5G,H) pathway analyses, revealed distinct temporal patterns of biological processes and signaling pathways in response to radiation. At IR1D, GO enrichment highlighted processes related to cellular stress responses, DNA damage repair, and apoptotic signaling, while KEGG analysis identified significant enrichment of the p53, MAPK, and TNF signaling pathways—core pathways involved in radiation-induced DNA damage, oxidative stress, and early inflammatory activation. At IR3D, GO enrichment shifted toward immune–inflammatory responses, leukocyte migration, and extracellular matrix remodeling, consistent with the progression of intestinal injury. Correspondingly, KEGG pathways were dominated by NF-κB signaling, Th1/Th2 cell differentiation, and cell adhesion molecule pathways, indicating sustained inflammatory activation and dysregulation of intestinal barrier function at the late stage post-irradiation.

Notably, these transcriptomic profiles are highly concordant with our earlier experimental observations. The early activation of stress and repair pathways at IR1D aligns with the acute induction of oxidative stress and the initial upregulation of P-gp expression, while the sustained enrichment of immune–inflammatory pathways at IR3D supports the hypothesis that chronic inflammation serves as a key driver of persistent P-gp upregulation and exacerbated intestinal barrier damage. Furthermore, the activation of cell cycle and DNA repair pathways, particularly at IR1D, reflects the adaptive compensatory responses of the small-intestinal epithelium to radiation-induced injury, attempting to maintain tissue homeostasis.

Collectively, these transcriptomic data provide a comprehensive, systems-level view of the dynamic molecular events occurring in the irradiated small intestine, identifying key signaling pathways that may mediate radiation-induced P-gp dysregulation and intestinal injury. These findings lay a robust foundation for future mechanistic investigations into the molecular regulation of drug transporters following radiation exposure, as well as for the development of targeted interventions to mitigate radiation-induced gastrointestinal toxicity.

### 2.6. Biochemical Markers of Intestinal Oxidative Stress and Activation of Related Pathway mRNAs Following Irradiation

Ionizing radiation triggers systemic oxidative stress [40]. We detected catalase (CAT) activity, glutathione (GSH) level, and superoxide dismutase (SOD) activity in mouse small intestine to evaluate oxidative stress, as these molecules form the core antioxidant defense against radiation-induced ROS [41]. Compared with the control group, intestinal CAT activity was significantly reduced at one day post-irradiation (IR-1d, *p* < 0.01) and further declined at 3 days (IR-3d, *p* < 0.001), showing a time-dependent decrease (Figure 6A). GSH level was also markedly lowered in IR-1d (*p* < 0.05) and further depleted in IR-3d (*p* < 0.01, Figure 6B). Similarly, SOD activity declined progressively after irradiation (*p* < 0.01 at IR-3d, Figure 6C). These results indicate that gamma-ray irradiation impairs antioxidant enzyme activity and exhausts non-enzymatic antioxidants, resulting in ROS accumulation and redox imbalance in the intestine.

We further examined the Nrf2/HO-1 pathway [42]. HO-1 mRNA showed a partially uncoupled temporal pattern from Nrf2 as a key Nrf2 target. HO-1 mRNA was significantly upregulated at IR-1d (1.6-fold, *p* < 0.05) and further increased at IR-3d (2.2-fold, *p* < 0.001), whereas Nrf2 mRNA was elevated early (IR-1d) but returned to low levels by IR-3d (Figure 6D,E). These findings indicate that gamma-ray-induced oxidative stress is associated with activation of the Nrf2-driven antioxidant pathway. While oxidative stress indicators, Nrf2/HO-1 expression, and *ABCB1/Abcb1a* mRNA upregulation strongly suggest a temporal correlation between Nrf2/HO-1 pathway activation and P-gp upregulation following irradiation, we acknowledge that this association does not establish causation. The concurrent activation of multiple stress-responsive pathways (including NF-κB and MAPK, as revealed by our transcriptomic analysis) may also contribute to P-gp regulation. Future studies employing targeted gene knockdown or pharmacological inhibition of Nrf2/HO-1 are necessary to definitively determine whether this pathway directly mediates radiation-induced P-gp upregulation.

## 3. Discussion

This study investigated the regulatory effects and mechanisms of different radiation conditions on P-gp, using gamma rays as the primary research subject. Combining cellular and animal experiments, it systematically explored the effects of radiation on P-gp from three dimensions: genes, proteins, and activity. The core results are largely consistent with the research hypotheses, revealing the potential temporal characteristics and molecular network of radiation-regulated P-gp.

Regarding the regulation of P-gp by gamma rays, this study observed an upward trend that is consistent with P-gp upregulation in its expression, and the upregulation exhibited a certain dose-dependent effect. In cellular experiments, after gamma-ray irradiation, the expression of *ABCB1* mRNA and P-gp protein fluctuated in different intestinal epithelial cells such as Caco-2 and IEC-6, as well as HK-2 and Beas-2b cells, suggesting that the response of P-gp to gamma rays is cellularly universal and not a specific response to a particular cell type. Radiation regulation of P-gp in different tissues may exhibit tissue specificity, which also suggests that radiation regulation of P-gp is complex and requires analysis considering specific tissue types and radiation conditions. Animal experiments further supported that after whole-body irradiation with 6 Gy gamma-rays, *Abcb1a* mRNA and P-gp protein in mouse small-intestinal tissue were significantly upregulated 1 and 3 days post-irradiation. Both the Transwell model and in vivo intestinal perfusion experiments showed a synchronous enhancement of P-gp transporter activity. This echoes the conclusions of previous clinical studies on radiation-induced changes in drug transporter expression. However, this study is the first to clearly define the specific time window for gamma-ray regulation of P-gp in normal small-intestinal tissue, supplementing experimental data on radiation-induced P-gp regulation in normal tissues rather than tumor tissues, and overcoming the limitation of existing studies that mostly focus on tumor radiotherapy and chemotherapy resistance.

Existing research mainly focuses on the relationship between radiation-induced upregulation of P-gp expression in tumor cells and multidrug resistance. Regarding research on radiation types, existing studies mainly focus on X-rays and gamma rays, and are mostly studies of single radiation types (Table 1). While these studies share the commonality of confirming the induction of P-gp by radiation, the core difference lies in the research subjects and perspectives: this study uses normal cells and animals as research subjects, focusing on the regulation of P-gp in normal tissues by radiation as an independent stressor, rather than the drug resistance mechanism under combined radiotherapy and chemotherapy in tumors. This fills the research gap on the regulation of drug transporters by radiation in normal tissues and is more consistent with the physiological state of normal radiation-exposed populations such as nuclear industry workers and radiotherapy patients.

Regarding regulatory mechanisms, existing studies mostly argue that signaling pathways such as NF-κB and AP-1 are involved in the regulation of radiation-induced P-gp expression [43,44]. Transcriptomic analysis provided key clues to the molecular mechanism of radiation-regulated P-gp. Results showed that one day post-irradiation, cellular stress, cell cycle arrest, and acute immune defense were the core themes, with significant enrichment of signaling pathways such as p53, MAPK, and TNF. Three days post-irradiation, the focus shifted to chronic immune inflammation activation and tissue repair, with significantly enhanced enrichment of pathways such as Th1/Th2 cell differentiation and NF-κB. This temporal change closely matches the expression pattern of P-gp, suggesting that radiation may indirectly regulate the transcription and expression of P-gp by activating the aforementioned signaling pathways. We clearly state that current qRT-PCR data only reflect steady-state mRNA levels, and we cannot distinguish enhanced transcription from prolonged mRNA half-life without promoter activity or RNA stability assays. Combined with transcriptomic enrichment of p53/NF-κB/Nrf2 stress transcription pathways and consistent mRNA-protein expression trends, we only tentatively infer transcriptional activation as the dominant driving factor. Subsequent experiments could incorporate mRNA stability assays and promoter-binding ChIP-qPCR for future mechanistic studies. Furthermore, existing studies on detecting P-gp activity after radiation primarily utilize in vitro cell models [45]. This study combines the Transwell model with in vivo intestinal perfusion experiments in mice to simultaneously detect P-gp activity in vitro and in vivo, making the experimental results more clinically valuable.

Although this study has achieved certain results, it still has some limitations and can be further improved in subsequent studies. First, regarding the radiation conditions, this study primarily used 6 Gy gamma rays as the animal radiation dose [28], without setting different dose gradients to explore the dose-response relationship between dose and P-gp regulation. This radiation did not allow a full investigation into the effects of different doses and multiple irradiation frequencies on animal-level P-gp. Second, the small intestine was studied as the main target tissue in animal experiments with respect to the expression and activity of P-gp. Changes in other tissues with high P-gp expression such as the liver have not yet been analyzed. However, human renal epithelial HK-2 and human lung epithelial Beas-2b cells were used in the study. Future research could comprehensively reveal the effects of radiation on the body’s drug transporter P-gp through multi-organ cell and tissue studies. The in vivo intestinal perfusion experiments were not performed with a P-gp inhibitor. Therefore, the observed decrease in P_eff_ may be partially attributable to radiation-induced mucosal barrier damage and reduced absorptive surface area, rather than solely to enhanced P-gp-mediated efflux. We have explicitly listed this as a limitation of our study and suggest that future investigations employ P-gp knockout mice or co-administration of a specific inhibitor such as zosuquidar via the intravenous route to further validate the in vivo contribution of P-gp. In addition, gamma-ray irradiation may exert dual chemical effects on P-gp protein. Ionizing radiation and radiation-induced reactive oxygen species (ROS) can directly oxidize amino acid residues, break peptide bonds, and disrupt the spatial conformation of P-gp, which may trigger protein denaturation and degradation [46,47]. On the other hand, excessive ROS also acts as a stress signal to activate downstream oxidative stress and inflammatory pathways [1]. Additionally, radiation-induced lipid peroxidation and post-translational modification (e.g., phosphorylation) may further regulate the functional activity of membrane-localized P-gp. Finally, regarding the exploration of molecular mechanisms, this study screened potential regulatory pathways and differentially expressed genes through omics analysis. However, the specific molecular mechanisms regulating P-gp were not clarified. Further experimental investigations can be conducted on key pathways and differentially expressed gene, and the role of epigenetic modifications (such as DNA methylation and miRNA regulation) can be explored [46,47,48,49]. While our results demonstrate a temporal correlation between Nrf2/HO-1 pathway activation and P-gp upregulation following irradiation, we acknowledge that this association does not establish causation. The concurrent activation of multiple stress-responsive pathways (including NF-κB and MAPK, as revealed by our transcriptomic analysis) may also contribute to P-gp regulation. Future studies employing targeted gene knockdown or pharmacological inhibition of Nrf2/HO-1 are necessary to definitively determine whether this pathway directly mediates radiation-induced P-gp upregulation. Furthermore, with the widespread application of nuclear technology, radiation exposure scenarios are becoming increasingly complex, and the risks of mixed radiation exposure, such as to gamma rays and neutron rays, are gradually increasing [50,51,52]. Future research could explore the regulatory effects of mixed radiation exposure on P-gp, providing experimental support for protection and rational drug use in complex radiation scenarios.

In summary, this study demonstrates that acute high-dose gamma-ray irradiation is associated with upregulation of intestinal P-gp expression and enhanced efflux activity in a mouse model. These findings provide an experimental basis for understanding drug transport changes under acute high-dose radiation exposure scenarios and may inform individualized pharmacotherapy considerations for populations exposed to such conditions. We acknowledge that the single 6 Gy whole-body irradiation used in this study represents an acute high-dose exposure scenario and is not directly comparable to occupational radiation exposure or conventional fractionated radiotherapy. The translational relevance of our findings is primarily to scenarios involving acute, high-dose radiation exposure, such as nuclear accidents and radiological emergencies, and potentially as an experimental model for understanding the mechanisms of radiation-induced transporter dysregulation. Additionally, the in vitro Transwell and in situ intestinal perfusion models established in this study may serve as useful platforms for future screening of drugs targeting radiation-regulated transporters. However, further studies using clinically relevant fractionated protocols or lower dose rates, as well as multi-organ assessments and mechanistic investigations including P-gp inhibitor or knockout validation, are needed to determine whether similar P-gp regulation occurs under more clinically applicable conditions and to establish evidence-based guidelines for precision pharmacotherapy in diverse radiation exposure settings. We therefore encourage readers to interpret our findings within the specific context of acute high-dose whole-body irradiation, and we emphasize that extrapolation to chronic or fractionated exposure contexts requires additional investigation.

**Table 1:** Comparison of selected previous studies on radiation-induced regulation of P-gp.

Irradiation Type/Dose	Model (Tumor/Normal Cells/Tissues)	Detected P-gp Changes	References
X-ray/The 10 Gy and 20 Gy groups received a dose of 2.0 Gy five times per week; the 50 Gy group received 2.0 Gy two days per week.	Human nasopharyngeal carcinoma cell line CNE1	P-gp protein expression correlates with MDR1 mRNA expression; the higher the irradiation dose, the higher the relative level of protein expression.	[37]
X-ray/LDFRT (0.5 Gy in 4 fractions at 6-h intervals); 2 Gy; 7 Gy	Oral squamous cell carcinoma cell lines T167 and T409	MDR1 expression in LDFRT was not altered by 2 Gy or 7 Gy radiation-induced expression.	[39]
X-ray/Total dose of 27 Gy	Brain tissue from 4–5-week-old male Wistar rats	P-gp content in the irradiated hemisphere decreased by nearly 40%.	[53]
X-ray/1 Gy	Human laryngeal carcinoma HEp-2 cells	Both the contribution of the Rhodamine 123 efflux rate in the cells and the resistance to vincristine increased.	[54]
Gamma-ray/Total dose of 27 Gy, 1.8 Gy five times per week.	Colon cancer cell lines	P-gp protein levels increased significantly by 19-fold.	[55]
Gamma-ray/8 Gy	Rat hepatocytes	P-gp gene cDNA expression was upregulated 1.5-fold after 6 h of irradiation.	[56]

## 4. Materials and Methods

### 4.1. Chemicals and Reagents

Cell culture media and trypsin-EDTA were obtained from Gibco; fetal bovine serum (FBS) was purchased from Excell; penicillin–streptomycin mixture, phenol red solution, Krebs–Henseleit buffer, and 0.9% normal saline were acquired from Procell (Wuhan, China); digoxin standard was supplied by Sigma-Aldrich (Shanghai, China). Additional reagents including acetonitrile, methanol, ethanol, isopropanol, and 4% paraformaldehyde fixative solution were also prepared for subsequent experimental procedures.

### 4.2. Cell Culture and Treatments

Caco-2 cell (Meilun Biotechnology, Dalian, China) and HK-2 cell (Pricella, Wuhan, China) were maintained in MEM (Gibco, SuZhou, China) supplemented with 10% FBS (ExcellBio, Suzhou, China) and 1% penicillin–streptomycin (EallBio, Beijing, China); Beas-2b cell (Pricella, Wuhan, China) and IEC-6 cell (EallBio, Beijing, China) were maintained in RPMI-1640 (Servicebio, Wuhan, China) and DMEM (Gibco, Suzhou, China) supplemented with 10% FBS and 1% penicillin–streptomycin. Four cell lines were incubated in a constant-temperature incubator with 5% CO_2_ at 37 °C. Subculturing was performed using 0.25% EDTA-trypsin. The cell radiation group was irradiated, while the control group was not exposed to radiation. Cells were irradiated at room temperature using a ^60^Co gamma-ray source. The source-to-sample distance was fixed at 1.5 m. The absorbed dose rate was 1.25 Gy/min. Cells were exposed to total doses of 5, 15, or 30 Gy. After exposure, the cells were transferred back to the previous culture conditions. Cell viability was assessed by MTT assay, and the detailed results are presented in Figure S6.

### 4.3. Animals and Treatment

C57BL/6J wild-type male mice, specific-pathogen-free (SPF), aged 8–12 weeks and weighing 18.0–22.0 g, were selected as the radiation subjects in this experiment, purchased from Beijing Vital River Laboratory Animal Technology Co., Ltd. (Beijing, China).

#### 4.3.1. Cultivation Environment and Irradiation Conditions

All mice were acclimatized for three days prior to the experiment and randomly assigned to three groups: a control group, a group 1 day after irradiation, and a group 3 days after irradiation, with eight mice in each group. Mice received whole-body irradiation using the same ^60^Co gamma-ray source at a dose rate of 0.85 Gy/min. The source-to-animal distance was 2 m, and mice were placed in a specially designed Plexiglas restrainer with ventilation hole. The total dose was 6 Gy, delivered as a single fraction. The control group was placed in the restrainer for an equivalent time without radiation exposure. Mice were fasted for 12 h prior to organ collection and were euthanized at the corresponding time points.

#### 4.3.2. In Situ Intestinal Perfusion

The animals were grouped into sets of eight and randomly assigned to three groups: a blank control group (Control group), a 1-day-post-irradiation group (IR-1d group), and a 3-day-post-irradiation group (IR-3d group). A Cobalt-60 source was utilized as the source of gamma radiation, and in vivo intestinal perfusion experiments were conducted on the mice at 1 and 3 days following irradiation. A 1% sodium pentobarbital solution was prepared using Krebs–Ringer buffer as the solvent, and the mice were anesthetized at a dosage of 60 mg/kg. The small-intestinal perfusate was formulated using Krebs–Ringer buffer (pH 7.4) containing 20 μM digoxin and 50 μg/mL phenol red. The concentration of digoxin was selected to remain below the P-gp saturation threshold to facilitate the observation of differences in transport; phenol red was included to correct for water secretion and absorption occurring during the perfusion process (reflecting the intrinsic barrier function of the small intestine).

The effective permeability P_eff_ serves as an indicator of a drug’s capacity to traverse the small-intestinal wall; it reflects the net permeability of the drug from the intestinal lumen side to the bloodstream side and is positively correlated with the net amount of drug absorbed [57]. The calculation formula is as follows:

(1) $$P_{\mathrm{eff}}=-Q\times \ln(C_{\mathrm{out}(\mathrm{corr})}/C_{\mathrm{in}})/A$$

(2) $$C_{\mathrm{out}(\mathrm{corr})}=C_{\mathrm{out}}\times \frac{{\mathrm{CPR}}_{\mathrm{in}}}{{\mathrm{CPR}}_{\mathrm{out}}}$$

In this context, P_eff_ represents the effective permeability (cm/min); C_out_ and C_in_ denote the digoxin concentrations in the outflow and inflow, respectively; C_out (corr)_ is the corrected outlet digoxin concentration, which corrected value is used directly in the P_eff_ equation; Q is the perfusion rate; the surface area (A) of the perfused intestinal segment is calculated as A = 2πr × L, where r is the intestinal radius (based on the average of three diameter measurements of the perfused segment) and L is the length of the perfused small intestine; and CPR_in_ and CPR_out_ represent the phenol red concentrations at the inlet and outlet, respectively.

Prior to commencing formal in vivo intestinal perfusion, each mouse was weighed; based on individual body weight, an appropriate volume of a prepared sodium pentobarbital solution was administered via intraperitoneal injection to induce a state of deep anesthesia. The proximal jejunum was selected as the perfusion segment; the proximal end (inlet) was connected to a peristaltic pump, while the distal end (outlet) was connected to a collection tube. The intestinal lumen was gently flushed to remove luminal contents using pre-warmed physiological saline. The peristaltic pump was then activated to perform perfusion at a constant flow rate of 0.12 mL/min, utilizing a digoxin perfusion solution prepared in Krebs–Ringer buffer; samples were collected into separate tubes every 10 min. Upon completion of the experiment, the length and inner diameter of the perfused intestinal segment were precisely measured. The phenol red concentration in the collected samples was determined by measuring absorbance at a wavelength of 560 nm using a UV spectrophotometer, which was subsequently used to calculate the water correction factor.

### 4.4. Real-Time Quantification Polymerase Chain Reaction (RT-qPCR) Studies

A certain amount of RNA extraction reagent (TRIzol) was added to the aforementioned tissue samples or cells, and then samples were homogenized. The lysate was centrifuged and the clear supernatant was obtained after chloroform was added. RNA was precipitated from the supernatant using isopropanol. The precipitate was washed with 75% ethanol–water to remove impurities, dried, and then re-dissolved in DEPC water to obtain total RNA. The RNA content was determined by measuring the optical absorbance at 260 nm using a NanoDrop 2000 spectrophotometer (Thermo Fisher Scientific, Inc., Waltham, MA, USA). Total RNA was subjected to reverse transcription to synthesize cDNA. The cDNA samples were mixed with custom primers and Taq Pro Universal SYBR qPCR Master Mix (Vayzme, Nanjing, China), then real-time fluorescence quantitative PCR was performed. Detailed reaction systems and protocols for reverse transcription and qPCR are listed in Tables S1–S4. The commercial Taq Pro Universal SYBR qPCR Master Mix we adopted is integrated with active DNase component, which can thoroughly degrade residual genomic DNA carried over from RNA samples throughout the qPCR setup and reaction. This one-step reagent design can avoid interference of genomic DNA on amplification results, so we omitted the independent DNase digestion procedure. Β-actin was used as a housekeeping gene, and the relative expression of the target gene was calculated using the 2^−∆∆Ct^ method. The coding sequence of the target gene was located using the National Center for Biotechnology Information (NCBI) database. The desired sequence was then designed using a primer design tool (primer-blast). In mouse tissues, RT-qPCR primers were specifically designed to target *Abcb1a*, the predominant P-gp isoform expressed in the murine intestinal epithelium. Finally, the specificity of the synthesized primers was verified using homologous cDNA samples. Primer sequence synthesis was provided by Sangon Biotech (Shanghai, China) Co., Ltd. The primer sequences are shown in Table S5.

### 4.5. Western Blotting

The protein levels of P-glycoprotein and beta-actin in cells and small-intestinal tissues were quantified via Western blot (Anti-P Glycoprotein, ab170904, Abcam, 1:1000 dilution; Beta Actin Monoclonal antibody, Proteintech, 66009-1-Ig, 1:1000 dilution). Horseradish peroxidase-conjugated secondary antibodies were used to bind to the primary antibodies (secondary antibodies: ZSGB, ZB-2301, 1:5000 dilution). A chemiluminescence detection system was employed to acquire signals from the labeled protein bands.

### 4.6. Immunofluorescence Staining

Cells were seeded onto sterile glass coverslips in 24-well plates. At sampling, cells were fixed with 4% paraformaldehyde for 15 min at room temperature, washed three times with PBS (5 min each), permeabilized with 0.3% Triton X-100 (EallBio, Beijing, China, catalog P0096) for 15 min, and blocked with 3% BSA (EallBio, Beijing, China, catalog 01.10001) for 1 h at room temperature. The coverslips were then incubated with the primary antibody (Anti-P Glycoprotein, Abcam ab170904, rabbit monoclonal, 1:200 dilution) overnight at 4 °C in a humidified chamber. After three washes with PBS (5 min each), the cells were incubated with Cy3-conjugated goat anti-rabbit IgG secondary antibody (Servicebio, Wuhan, China, catalog GB21303, 1:300 dilution) for 50 min at room temperature in the dark. Nuclei were counterstained with DAPI solution (10 μg/mL, EallBio, Beijing, China, catalog 02.10539) for 10 min at room temperature in the dark. After three additional washes with PBS (5 min each), the coverslips were mounted onto glass slides using anti-fade mounting medium (Solarbio, Beijing, catalog S2110). Negative controls were prepared by omitting the primary antibody incubation; these consistently showed no detectable fluorescence signal, confirming antibody specificity. Images were acquired using an OLYMPUS DP26 microscope at 400 × magnification. Cy3 fluorescence was captured using a standard Cy3 filter cube (excitation 510–560 nm, emission 570–620 nm). The acquired Cy3 signal was pseudocolored green for presentation, while nuclei visualized in the DAPI channel were pseudocolored blue. P-gp was visualized using a Cy3-conjugated secondary antibody, with images acquired through the green fluorescence channel (pseudocolored green for presentation), while nuclei were visualized in the DAPI channel (blue fluorescence). Quantitative fluorescence intensity analysis was performed using ImageJ software (version 1.54m; National Institutes of Health, Bethesda, MD, USA), with background correction applied to all images.

### 4.7. LC–MS/MS Conditions

The separation column employed was ACQUITY UPLC BEH C18 1.7 μm, and the instrument model was API4000. Electrospray ionization (ESI) in positive ion mode, coupled with multiple reaction monitoring (MRM) scanning mode, was utilized to achieve highly specific detection of digoxin and the internal standard, digitoxin. The precursor/product ion pair for digoxin was 798.4/651.4, and for the internal standard digitoxin, it was 782.4/635.4; the collision energy (CE) was uniformly set to 20.0 V, and the collision gas (CAD) pressure was 6. The ion source parameters were as follows: ion spray voltage 5500 V, source temperature 350 °C, declustering potential (DP) 100.0 V, entrance potential (EP) 10.0 V, and collision cell exit potential 10.0 V. With digitoxin serving as the internal standard, a weighted linear regression model (1/x^2^ weighting) was employed to fit the digoxin standard curve.

### 4.8. Construction and Validation of the Transwell Dense Intestinal Epithelial Cell Model

Vigorously growing Caco-2 cells were harvested and seeded onto Transwell polycarbonate membranes at a density of approximately 6000 cells/cm^2^. The cells were maintained under the same culture conditions as the parent Caco-2 cells; once a confluent monolayer had formed, the integrity of the barrier was verified by measuring the transepithelial electrical resistance (TEER) across the inner and outer chambers, while permeability was assessed via a fluorescein transport assay. Upon validation of the model, substrate drug transport experiments were conducted.

The disparity in transport concentrations was quantified using the efflux ratio (ER), calculated according to the following formula:

(3) $$\mathrm{ER}=P_{\mathrm{app}}(B\to A)/P_{\mathrm{app}}(A\to B)$$

(4) $$P_{\mathrm{app}}=(\Delta Q/\Delta t)/(AC_{0})$$

In this equation, P_app_ represents the apparent permeability coefficient (cm/s); ΔQ is the amount of drug permeating through the monolayer during the time interval Δt; A is the diffusion area (cm^2^); and C_0_ is the initial drug concentration in the donor compartment. In the ER formula, Side A corresponds to the apical side, simulating the intestinal lumen; Side B corresponds to the basolateral side, simulating the bloodstream. The direction A→B represents absorptive transport (simulating drug absorption from the intestinal lumen into the blood), while the direction B→A represents efflux transport (simulating drug efflux from the blood back into the intestinal lumen [58]).

#### 4.8.1. Transepithelial Electrical Resistance

The transepithelial electrical resistance (TEER) values were measured using an STX2 chopstick electrode with EVOM2™ (World Precision Instruments, Sarasota, FL, USA), following the manufacturer’s instructions, to evaluate cell layer integrity. The measurements obtained were corrected by subtracting the empty insert value and multiplying it by the surface area of the culture insert (0.33 cm^2^) to derive the final TEER values [59].

(5) $$TEER(\Omega \cdot {\mathrm{cm}}^{2})=[TEER(\Omega )-TEER_{\mathrm{Blank}}(\Omega )]\times A({\mathrm{cm}}^{2})$$

#### 4.8.2. Fluorescent Yellow Translucency

Permeability experiments were conducted using fluorescein CH dipotassium salt (Fluorescein Yellow) powder. The powder was dissolved in HBSS buffer solution to prepare a working solution with a concentration of 10 μg/mL. A two-well chamber model was selected as the test system, and the prepared fluorescein working solution was added separately to the apical side and basolateral side of the two wells. After incubation in a constant-temperature incubator at 37 °C for 30 min, observations were performed under a fluorescence microscope; differences in permeability were assessed by comparing the fluorescence intensities observed under the microscope. Fluorescence intensity was quantified using ImageJ software (version 1.54m; National Institutes of Health, Bethesda, MD, USA). For each image, the mean fluorescence intensity was measured from at least three randomly selected fields per well. Background fluorescence was subtracted using a region of interest outside the cell monolayer. Relative fluorescence intensity was calculated by normalizing the values to the control group.

### 4.9. Determination of Total Bile Acid Levels in Mouse Serum

The CheKineTM Total Bile Acid (TBA) Content Assay Kit was used, and experimental procedures and result calculation were determined in accordance with the protocols provided by the manufacturer (Solarbio, Beijing, China). The samples consisted of serum from each mouse from different groups, which obtained and centrifuged at 3000 rpm for 5 min.

### 4.10. Tissue Immunohistochemistry (IHC)

The small intestines were washed with cold PBS, fixed in 4% paraformaldehyde, and embedded in paraffin. Sections (5 μm) were stained with HE or IHC. IHC started with antigen retrieval followed by overnight incubation with primary antibodies at 4 °C. Antibodies used included P-glycoprotein (Servicebio, GB115178), occludin (Servicebio, GB111401), and ZO-1 (Servicebio, GB15195). After incubation, secondary antibodies conjugated to horseradish peroxidase were applied (Servicebio, G1267-200T), and DAB staining was performed.

### 4.11. Detection of CAT, GSH, and SOD in Small-Intestinal Tissue

The concentrations of glutathione (GSH, Solarbio, BC1175), superoxide dismutase (SOD, Solarbio, BC5165), and catalase (CAT, Solarbio, BC0205) were determined in accordance with the protocols provided by the manufacturer. After the extract was added to the small-intestine tissue, it was homogenized on ice and centrifuged at 8000 *g* for 10 min at 4 °C. The resulting supernatant was utilized to assess the levels of glutathione (GSH), superoxide dismutase (SOD), and catalase (CAT) in intestinal tissue based on the manufacturer’s instructions (Solarbio, Beijing, China).

### 4.12. RNA Sequencing and Data Analysis

For RNA sequencing, 8 biological replicates per group were used. Total RNA was extracted from mouse small-intestinal tissues using TRIzol^®^ Reagent (Invitrogen, Carlsbad, CA, USA), quantified with a NanoDrop 2000 spectrophotometer (Thermo Fisher Scientific, Wilmington, DE, USA), and assessed for integrity using an Agilent 5300 Fragment Analyzer (Agilent Technologies, Santa Clara, CA, USA) (RIN ≥ 8.0). mRNA was purified using oligo (dT) magnetic beads, fragmented to 300 bp, reverse-transcribed into cDNA, end-repaired, A-tailed, ligated to adapters, size-selected (300–400 bp) by magnetic beads, and PCR-amplified to construct libraries, which were sequenced on an Illumina NovaSeq X Plus platform (PE150). Raw reads were quality-filtered with fastp (v0.23.4), and clean reads were aligned to the mouse reference genome (GRCm39) using HISAT2 (v2.2.1). Gene-level counts were quantified with featureCounts (v2.0.6), and transcript abundances were normalized as TPM using RSEM (v1.3.3). Differential expression analysis (DESeq2, v1.36.0, |log_2_FC| ≥ 1, FDR < 0.05), PCA, and sample correlation heatmaps were performed in R (v4.2.0). GO and KEGG functional enrichment analyses were conducted using clusterProfiler (v4.8.2) with an FDR < 0.05 cutoff.

### 4.13. Statistical Analysis

All experimental data were statistically analyzed using GraphPad Prism 8 software and expressed as mean ± standard deviation (Mean ± S.D.). For experiments involving two factors (radiation dose and time), two-way ANOVA with Tukey’s post hoc test for multiple comparisons was employed. For single-factor experiments, one-way ANOVA with Dunnett’s post hoc test was used for comparisons against the control group. Comparisons between two groups were performed using unpaired two-tailed Student’s *t*-tests. In all cases, n represents the number of independent biological replicates, not technical replicates. Specifically: for cell experiments, n = 3 represents three independent experiments performed on different days with different cell passages; for animal experiments, n = 8 represents eight individual mice. Technical replicates were averaged within each biological replicate before statistical analysis. *p* < 0.05 was considered statistically significant (* *p* < 0.05, ** *p* < 0.01, *** *p* < 0.001).

## Figures and Tables

**Figure 1:**
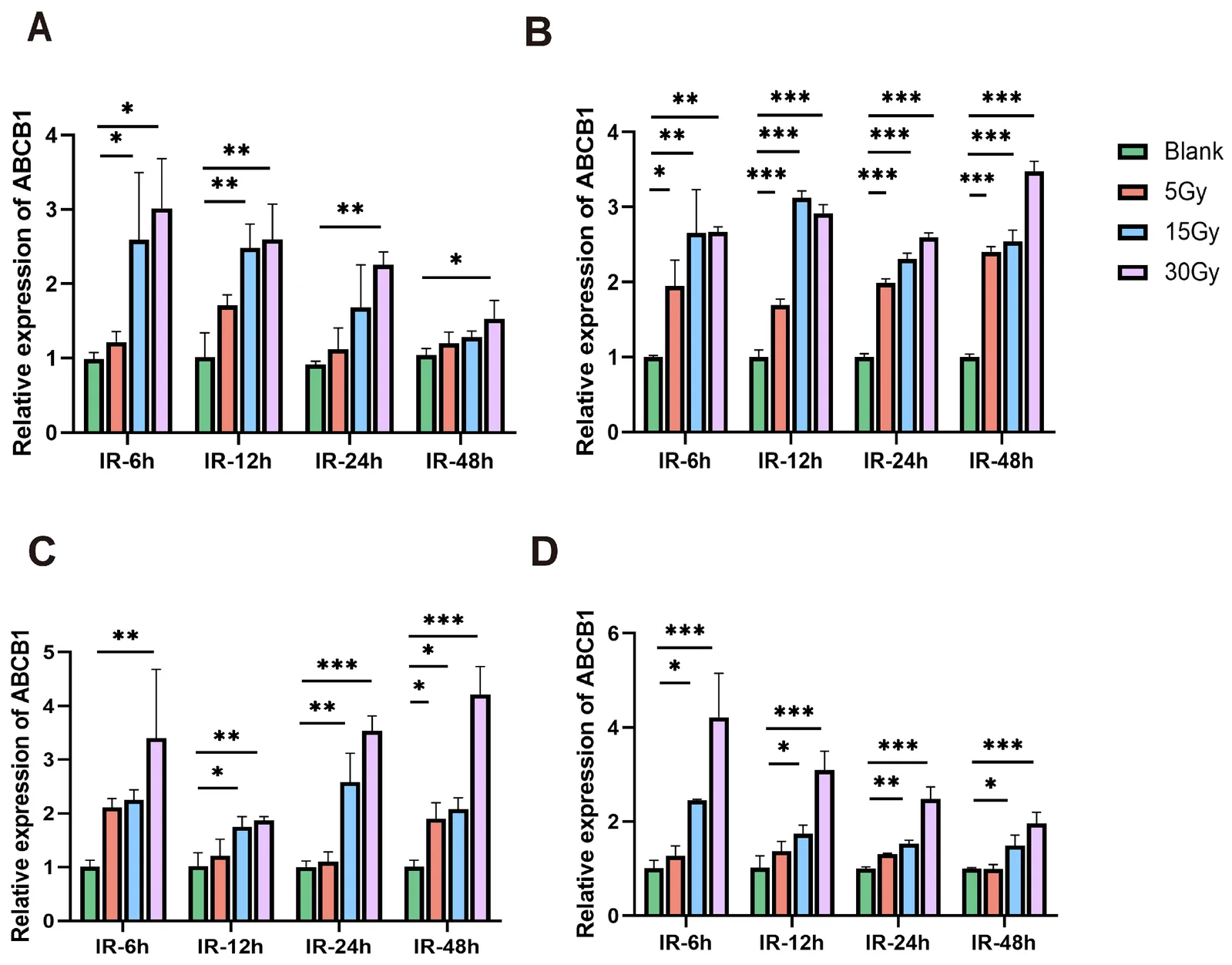
Expression of P-gp gene mRNA at different time points after gamma-ray irradiation in four cell lines. The relative expression of P-gp-related gene *ABCB1* mRNA in the 5 Gy, 15 Gy, and 30 Gy radiation dose groups compared to the control group (no radiation) of Caco-2 (**A**), IEC-6 (**B**), HK-2 (**C**), and Beas-2b (**D**). (Mean ± SD, *n* = 3, * indicates the control group compared, * *p* < 0.05, ** *p* < 0.01, *** *p* < 0.001).

**Figure 2:**
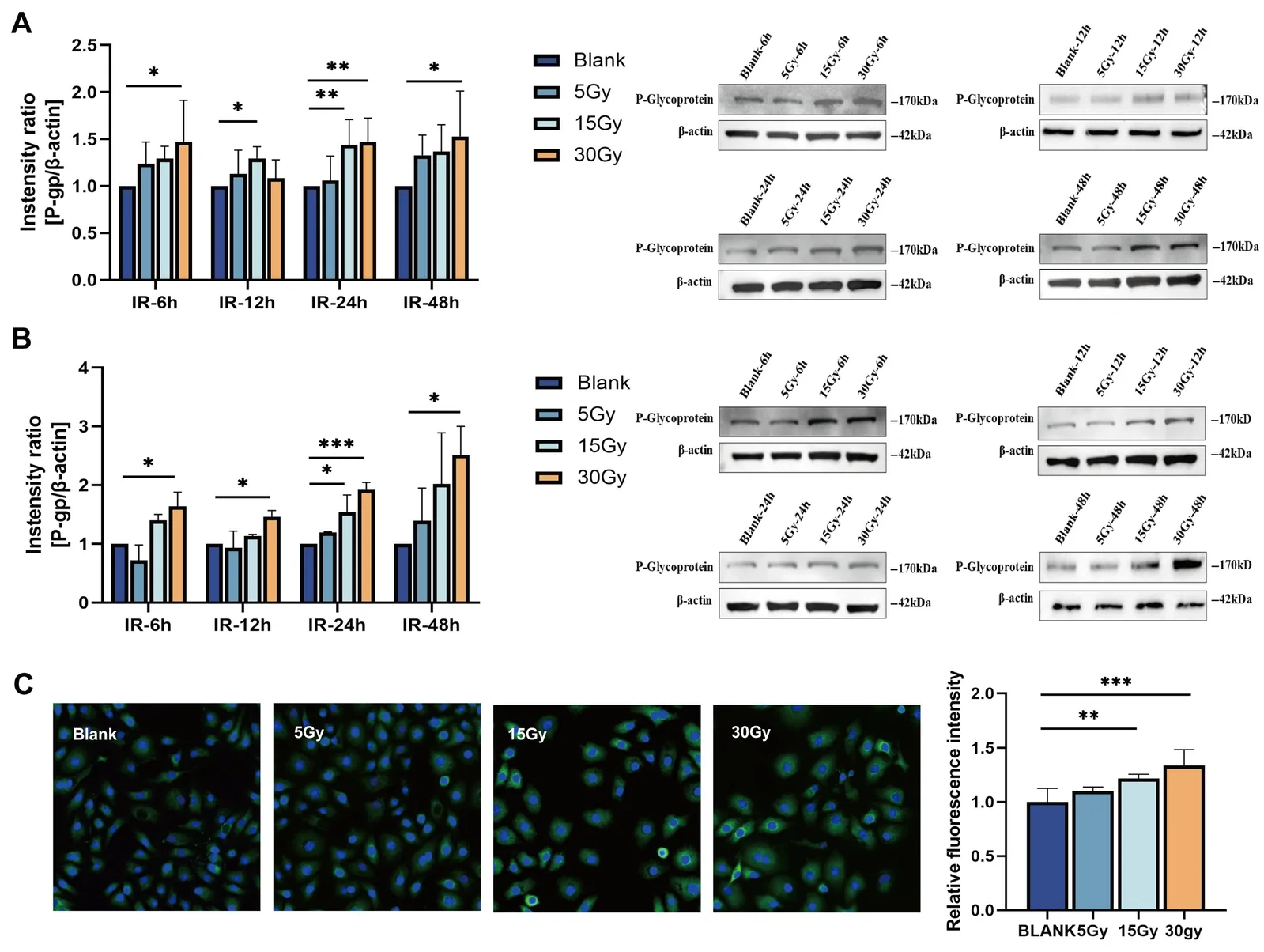
Relative P-gp protein expression after γ-ray irradiation with different doses (5, 15, 30 Gy) at sequential time points. (**A**) Quantification and original Western blot bands of P-gp/β-actin intensity ratio in irradiated Caco-2 cells at 6, 12, 24, and 48 h post-exposure. (**B**) P-gp protein abundance detected by Western blot in IEC-6 cells at the same four time points post-irradiation. (**C**) Representative immunofluorescence micrographs and quantitative relative fluorescence intensity of P-gp in IEC-6 cells. P-gp was detected using a Cy3-conjugated secondary antibody which was pseudocolored green for presentation. Nuclei were counterstained with DAPI and pseudocolored blue. The bar graph summarizes the statistical quantification of relative P-gp fluorescence intensity. (Mean ± SD, *n* = 3, * indicates the control group compared, * *p* < 0.05, ** *p* < 0.01, *** *p* < 0.001).

**Figure 3:**
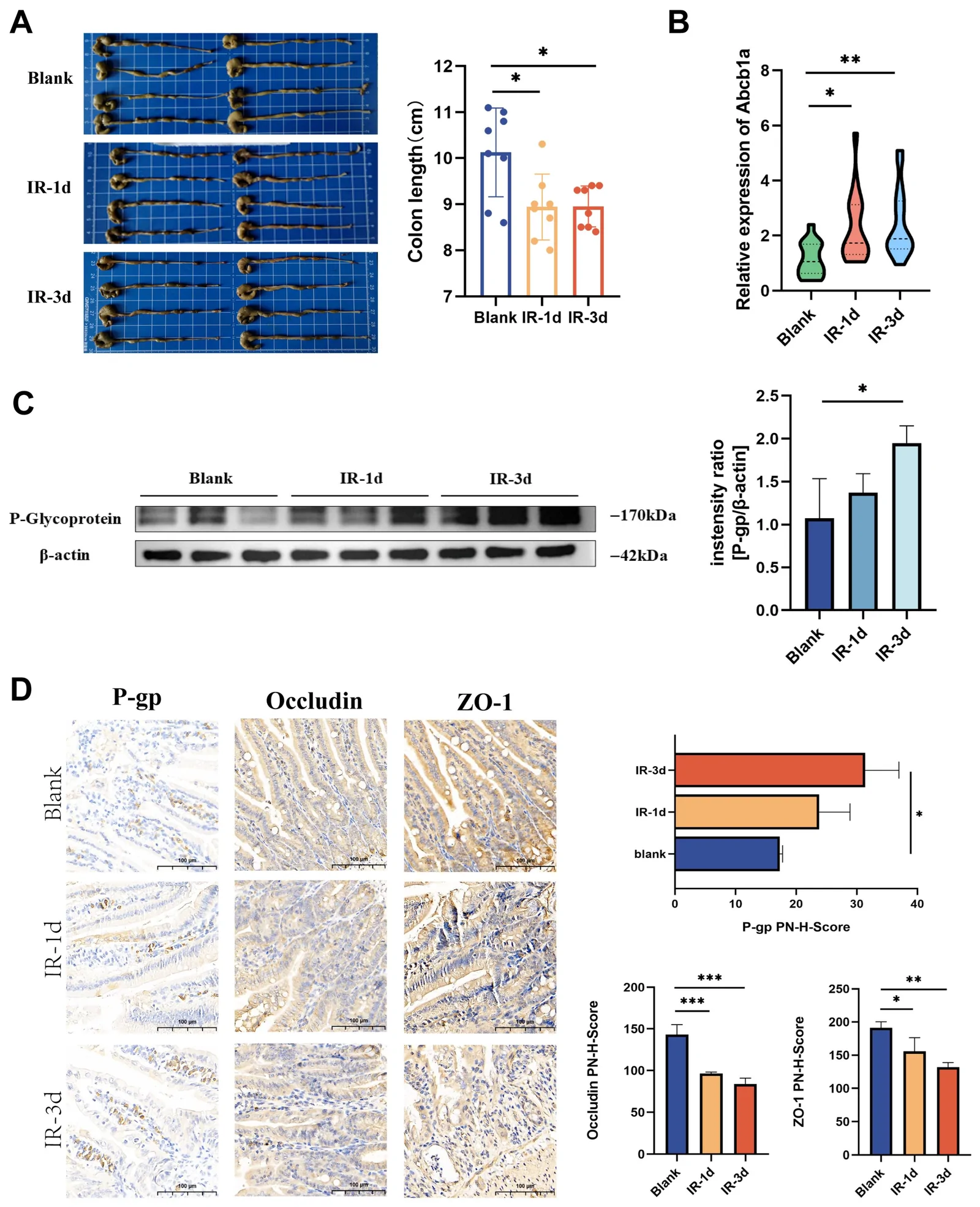
Effects of gamma-ray irradiation on mouse phenotypes and expression of *Abcb1a* gene and P-gp protein in small-intestinal tissue. (**A**) Changes in colon length in mice. (**B**) Relative mRNA expression level of the P-gp encoding gene (*Abcb1a*) in mouse small-intestinal tissue. (**C**) Relative expression levels of P-gp protein in mouse small-intestinal tissue (using β-actin as an internal reference). (**D**) Immunohistochemical staining of mouse small-intestinal tissue after irradiation; the target proteins were P-gp, occludin, and ZO-1; semi-quantitative analysis of immunohistochemical images using PN-H-Score. For IHC staining: brown/yellow granular signals represent positive immunoreactivity of target proteins (P-gp, occludin, ZO-1); the blue background corresponds to hematoxylin counterstaining of cell nuclei. A 100 μm scale bar is marked on each micrograph. (Mean ± SD, *n* = 8, * indicates the control group compared, * *p* < 0.05, ** *p* < 0.01, *** *p* < 0.001).

**Figure 4:**
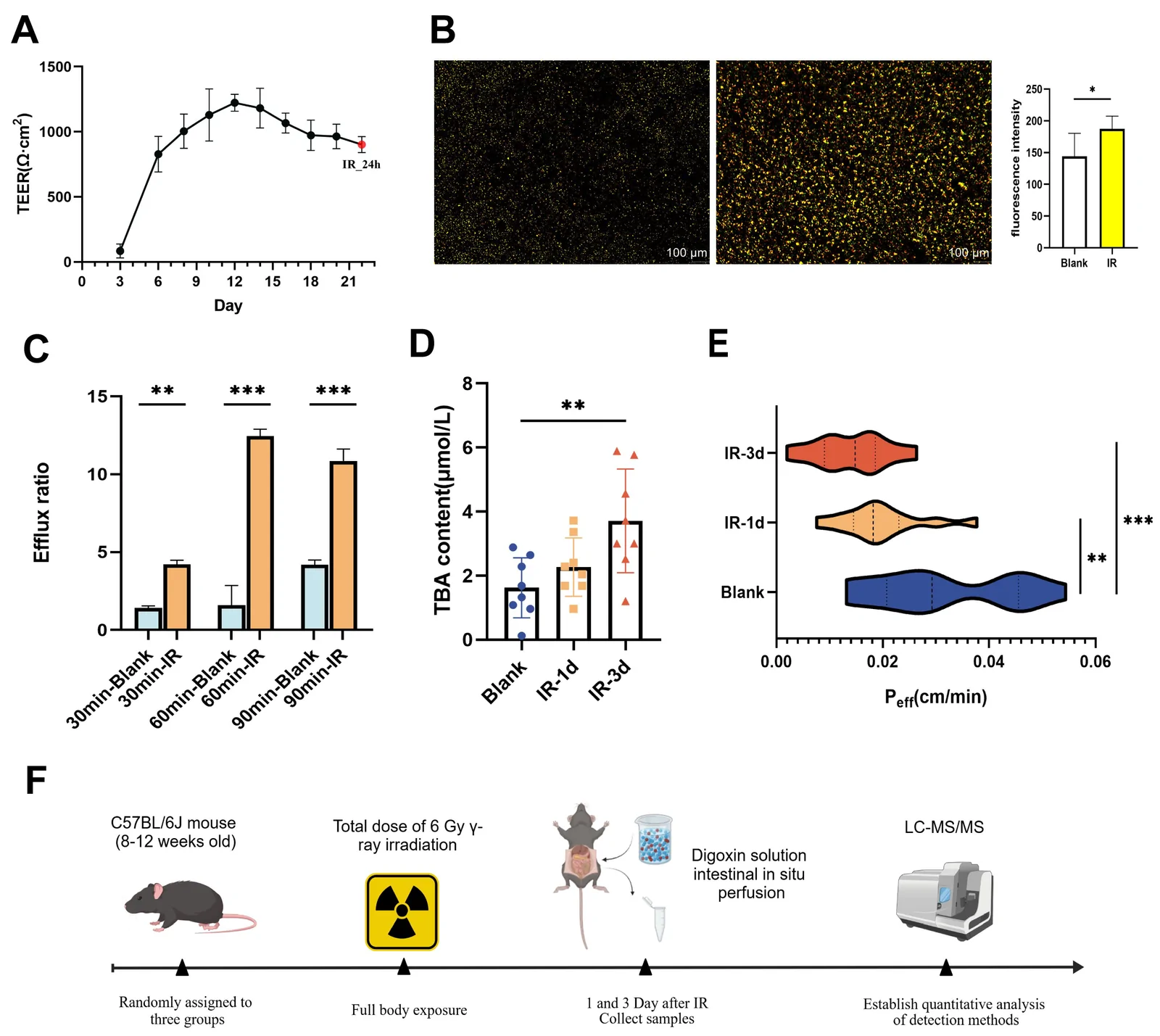
Experiments on the transport activity of P-gp transporters before and after irradiation. (**A**) Changes in TEER values after cell seeding in the Transwell chamber model (*n* = 6). (**B**) Fluorescent yellow permeability experiment using a Transwell chamber model; left image: unirradiated group; middle image: 15 Gy irradiated group; right image: quantitative fluorescence intensity data analyzed by ImageJ (version 1.54m; National Institutes of Health, Bethesda, MD, USA). (*n* = 3). (**C**) Changes in digoxin efflux rate in a Transwell intestinal epithelial barrier model of Caco-2 cells after radiation exposure (*n* = 3). (**D**) Changes in serum total bile acid concentration in mice (*n* = 8). (**E**) Changes in effective permeability of the small intestine in mice after radiation (n = 8). (**F**) In vivo transport activity assay of P-gp substrate drugs. (Mean ± SD, * indicates the control group compared, * *p* < 0.05, ** *p* < 0.01, *** *p* < 0.001).

**Figure 5:**
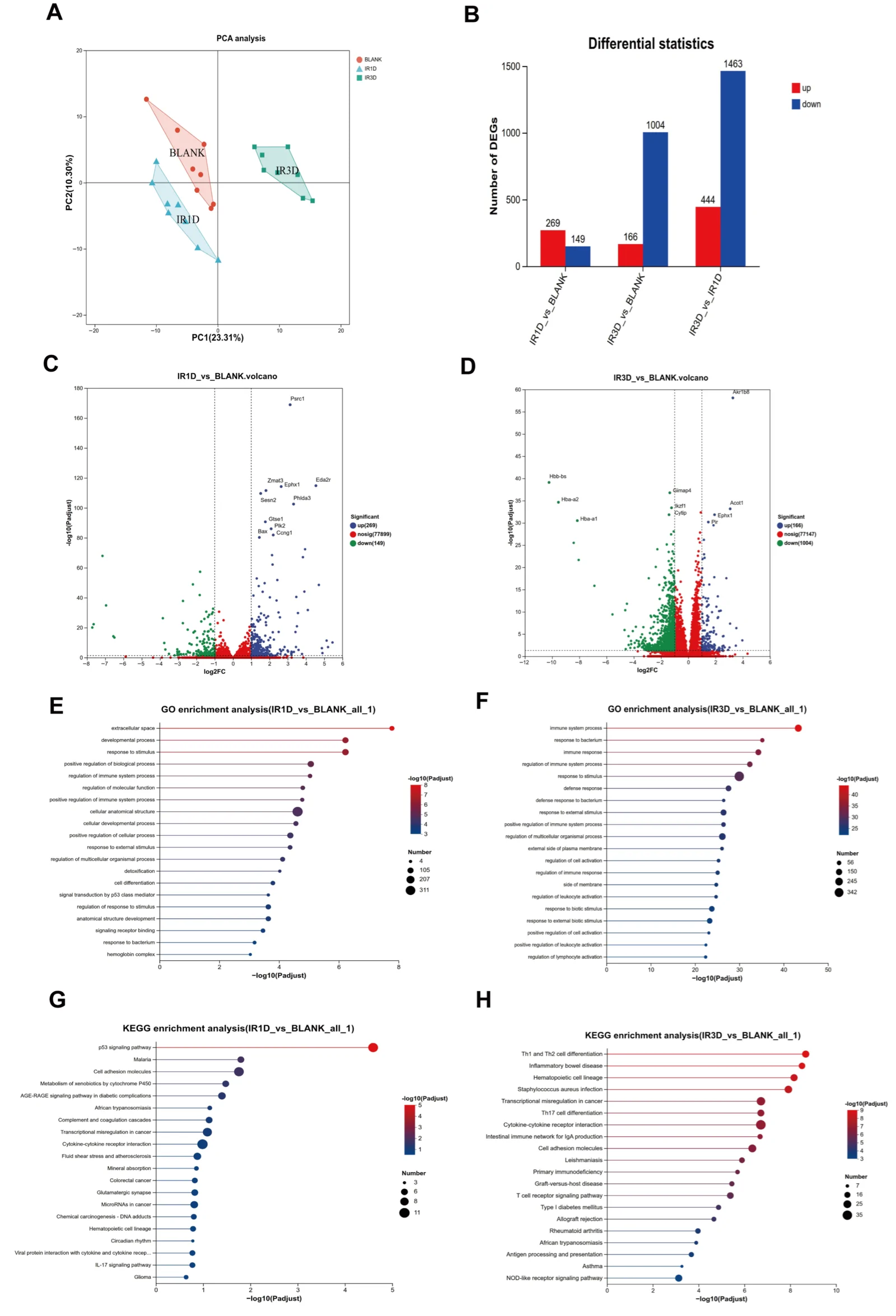
Transcriptomic study and analysis of small-intestinal tissue from radiation-induced mice (*n* = 8). (**A**) Principal component analysis (PCA). (**B**–**D**) Statistical analysis of differentially expressed genes in the small-intestine tissue of irradiated mice. (**E**–**H**) GO, KEGG differential gene functional enrichment analysis.

**Figure 6:**
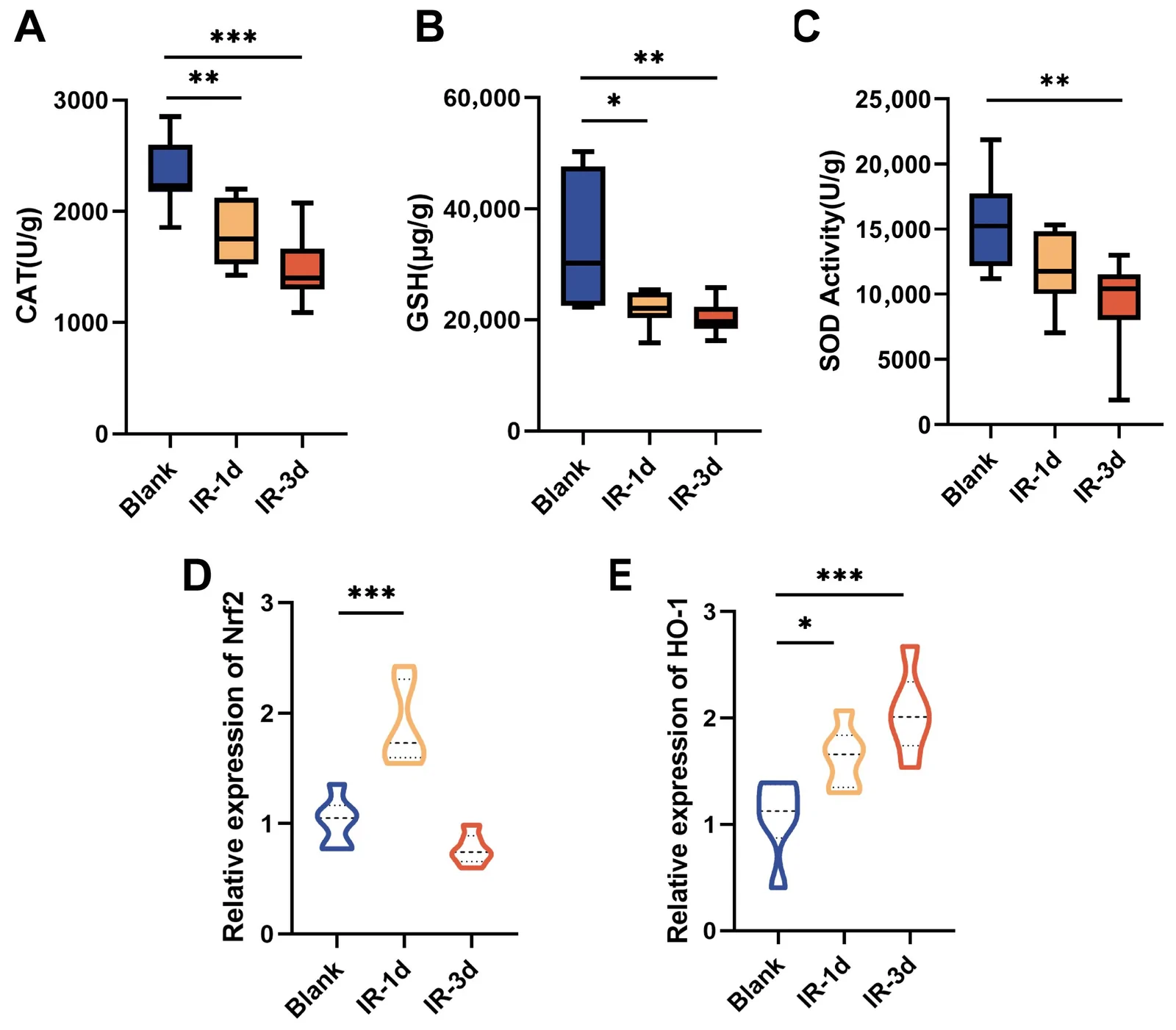
Biochemical markers of intestinal oxidative stress and activation of related pathway mRNAs following irradiation (n = 8). (**A**–**C**) Effect of irradiation with 6 Gy gamma-ray on concentration of CAT activity (**A**), GSH level (**B**), and SOD activity (**C**) in mouse small-intestine tissue. (**D**,**E**) Relative mRNA expression of Nrf2/β-actin (**A**) and HO-1/β-actin (**B**) in mouse small-intestine tissue after irradiation. (Mean ± SD, *n* = 8, * indicates the control group compared, * *p* < 0.05, ** *p* < 0.01, *** *p* < 0.001).

## Data Availability

The original contributions presented in this study are included in the article/Supplementary Material. Further inquiries can be directed to the corresponding author.

## Supplementary Materials

The following supporting information can be downloaded at: https://www.mdpi.com/article/10.3390/ijms27198586/s1.

## Abbreviations

The following abbreviations are used in this manuscript:

P-gp	P-glycoprotein
ABC	ATP-binding cassette
*A**BCB1*	ATP-binding cassette subfamily B member 1
ADME	Absorption, distribution, metabolism, and excretion
ARS	Acute radiation syndrome
Caco-2	Caucasian colon adenocarcinoma-2
CAD	Collision-activated dissociation
CAT	Catalase
CE	Collision energy
DAPI	4’,6-diamidinyl-2-phenylindole
DEGs	Differentially expressed genes
DMEM	Dulbecco’s modified eagle medium
DP	Declustering potential
DSBs	DNA double-strand breaks
EP	Entrance potential
ER	Efflux ratio
ESI	Electrospray ionization
FBS	Fetal bovine serum
GO	Gene Ontology
GSH	Glutathione
HBSS	Hank’s Balanced Salt Solution
HK-2	Human kidney-2
HO-1	Heme oxygenase-1
IEC-6	Intestinal epithelial cell line 6
IHC	Immunohistochemistry
IR	Irradiation
KEGG	Kyoto Encyclopedia of Genes and Genomes
LC-MS/MS	Liquid chromatography–tandem mass spectrometry
MRM	Multiple reaction monitoring
Nrf2	Nuclear factor erythroid 2-related factor 2
P_app_	Apparent permeability coefficients
PBS	Phosphate-buffered saline
PCA	Principal component analysis
P_eff_	Effective permeability
qRT-PCR	Real-time quantification polymerase chain reaction
SOD	Superoxide dismutase
SPF	Specific-pathogen-free
TBA	Total bile acid
TEER	Transepithelial electrical resistance
ZO-1	Zonula occludens-1
