# Supplementary material for: Regulation of Intestinal P-Glycoprotein by Gamma-Ray Irradiation: Impacts on Transporter Expression and Oxidative Stress
Source: Int J Mol Sci. 2026 Sep 25;27(19):8586. doi: 10.3390/ijms27198586 (PMC13664807; doi:10.3390/ijms27198586)
Supplement: Supplementary file 1 [file ijms-27-08586-s001.zip › ijms-4446208-supplementary.pdf]

Supplementary materials

Supplementary tables

Table S1. mRNA reverse transcription system

Experimental reagents	reagent volume
RNnase-free ddH_2O	To 20 μL
All-in-one qRT SuperMix Multifunctional enzymes	4 μL
Enzyme Mix	1 μL
template RNA	Total RNA:1 pg-1 μg

Table S2. mRNA reverse transcription procedure

Experimental temperature	Experimental time
50℃	15 min
85℃	5 s

Table S3. Quantitative Real-time PCR (SYBR Green) reaction system

Experimental reagents	reagent volume
Taq Pro Universal SYBR qPCR Master Mix	10.0 μL
Primer1（10 μM）	0.4 μL
Primer2（10 μM）	0.4 μL
Template cDNA	×μL
ddH_2O	To 20.0 μL

Table S4. Quantitative Real-Time PCR (SYBR Green) reaction procedure

Stage1	Pre-denaturation	Reps:1	95℃	30 sec
Stage2	cyclic reaction	Reps:40	95℃	10 sec
			60℃	30 sec
Stage3	melting curve	Reps:1	95℃	15 sec
			60℃	60 sec
			95℃	15 sec

Table S5. Sequence of primers used for qRT-PCR

Primers	Source		Sequence（5‘-3’）
Abcb1	Homo sapiens	Forward	CCCATCATTGCAATAGCAGG
		Reverse	GTTCAAACTTCTGCTCCTGA
β-actin	Homo sapiens	Forward	AGCCTTCCTTCCTGGGCAT
		Reverse	GCCAGGGCAGTGATCTCCTT
Abcb1a	Mus musculus	Forward	TCCTCACTAAGCGACTCCGA
		Reverse	GACCCTGTAGCCCCTTTCAC
Abcb1a	Rattus norvegicus	Forward	TGACACCCCTGAAATCCAGC
		Reverse	TGCCAAATGTGAAGCCCTGA
Nfe2l2	Mus musculus	Forward	GACTACAGTCCCAGCAGAGTG
		Reverse	TCTGCGTGTGTCTCAGAAACCTC
Hmox1	Mus musculus	Forward	CAGAGCCGTCTCGAGCATAG
		Reverse	AAATCCTGGGGCATGCTGTC
β-actin	Mus musculus	Forward	ATATCGCTGCGCTGGTCG
		Reverse	TTCCCACCATCACACCCTGG
β-actin	Rattus norvegicus	Forward	AGATCAAGATCATTGCTCCTCCT
		Reverse	ACGCAGCTCAGTAACAGTCC

Table S6. Data on digoxin transport in the Transwell intestinal epithelial model

time	Groups	Transit direction	Sampling concentration （ng/mL）	P_{app}	ER
30min	Blank	A→B	49.62	2.14E-05	1.41
		B→A	69.93	3.02E-05	
	IR(15Gy)	A→B	24.88	1.07E-05	4.21
		B→A	104.8	4.52E-05	
60min	Blank	A→B	92.28	1.99E-05	3.03
		B→A	279.61	6.03E-05	
	IR(15Gy)	A→B	26.55	5.72E-06	12.44
		B→A	330.32	7.12E-05	
90min	Blank	A→B	96.47	1.39E-05	4.19
		B→A	403.94	5.81E-05	
	IR(15Gy)	A→B	45.42	6.53E-06	10.86
		B→A	493.05	7.09E-05	

Supplementary figures

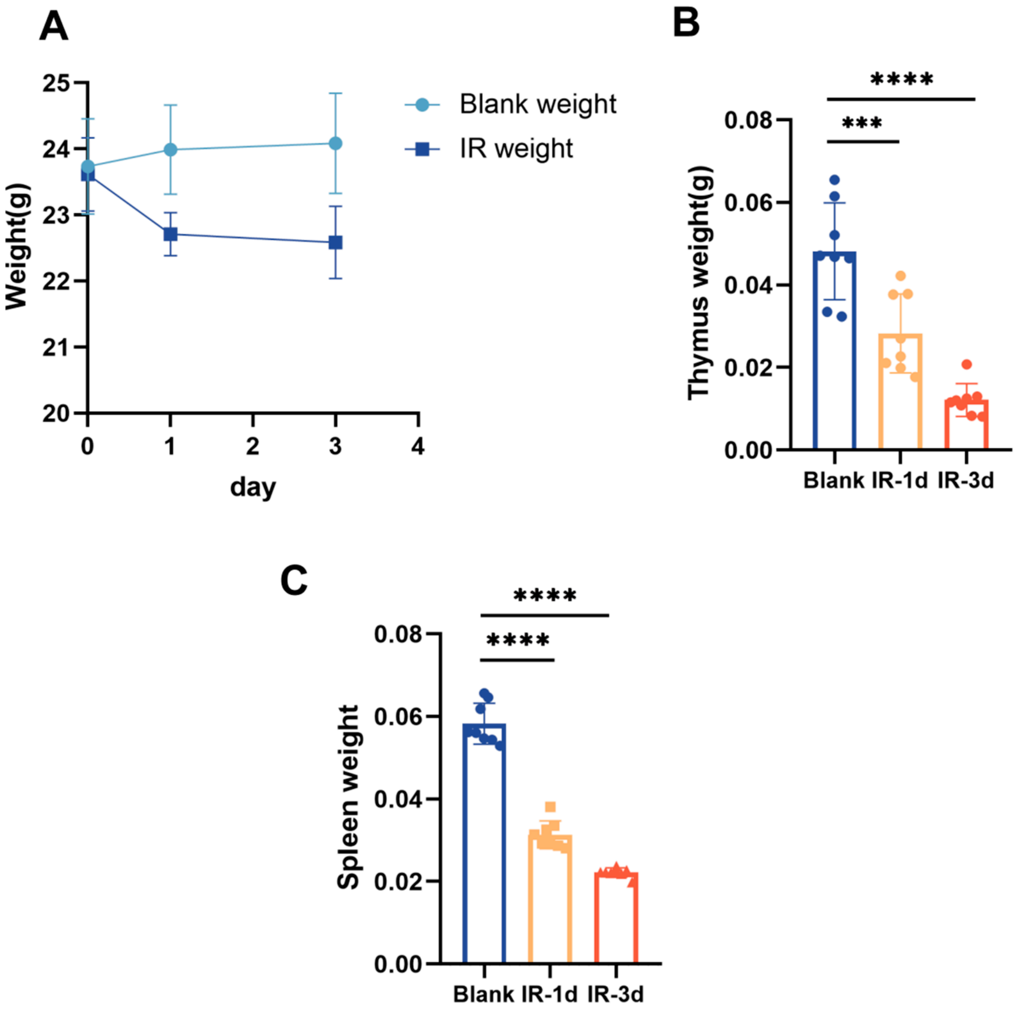

Figure S1. Organ index and body weight changes in mice of corresponding groups (n=8)

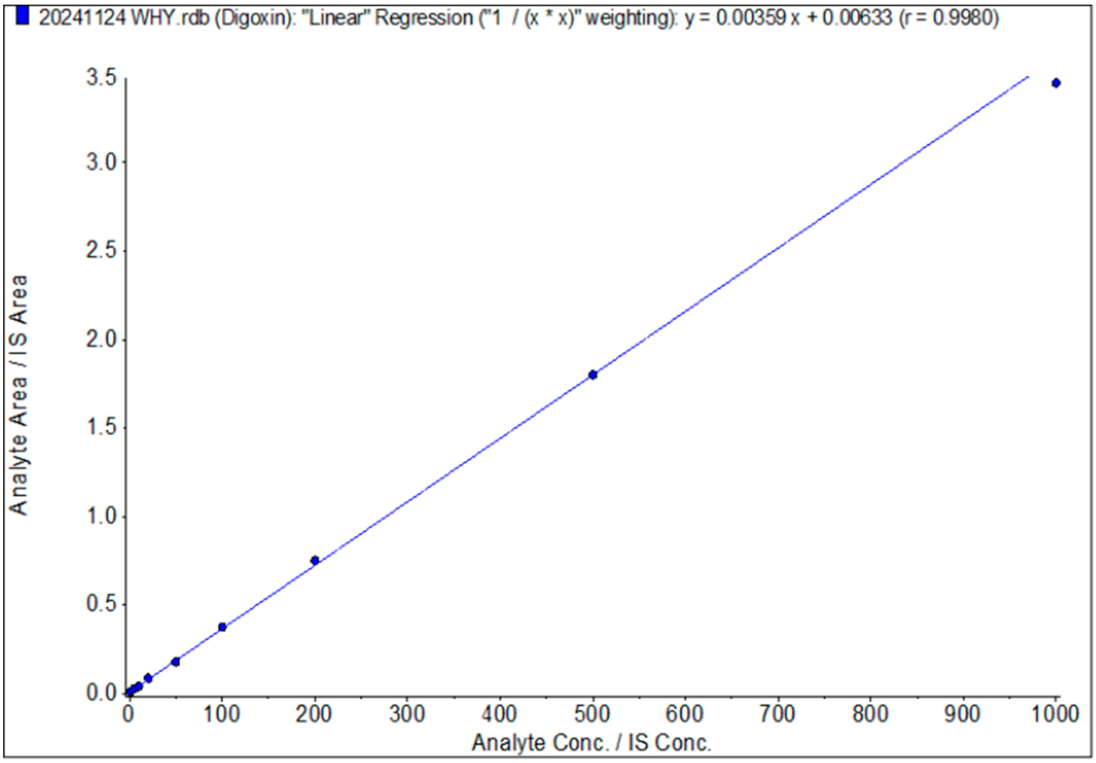

Figure S2. Digoxin standard curve in Transwell intestinal epithelial barrier model

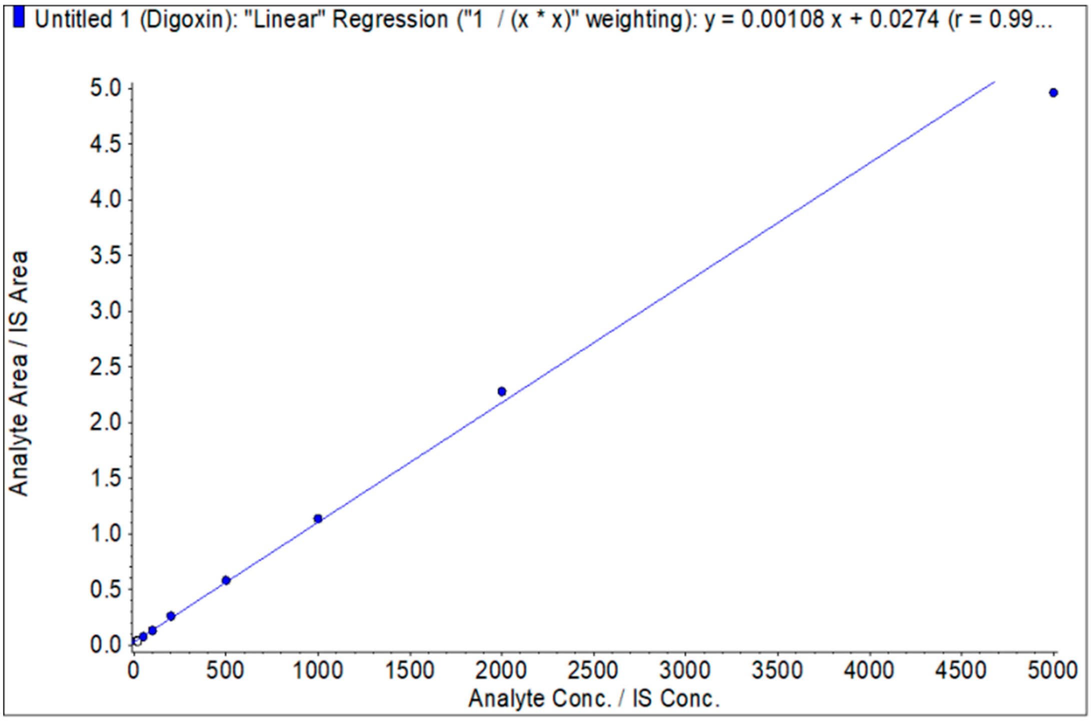

Figure S3. Digoxin standard curve of mouse in vivo intestinal perfusion samples

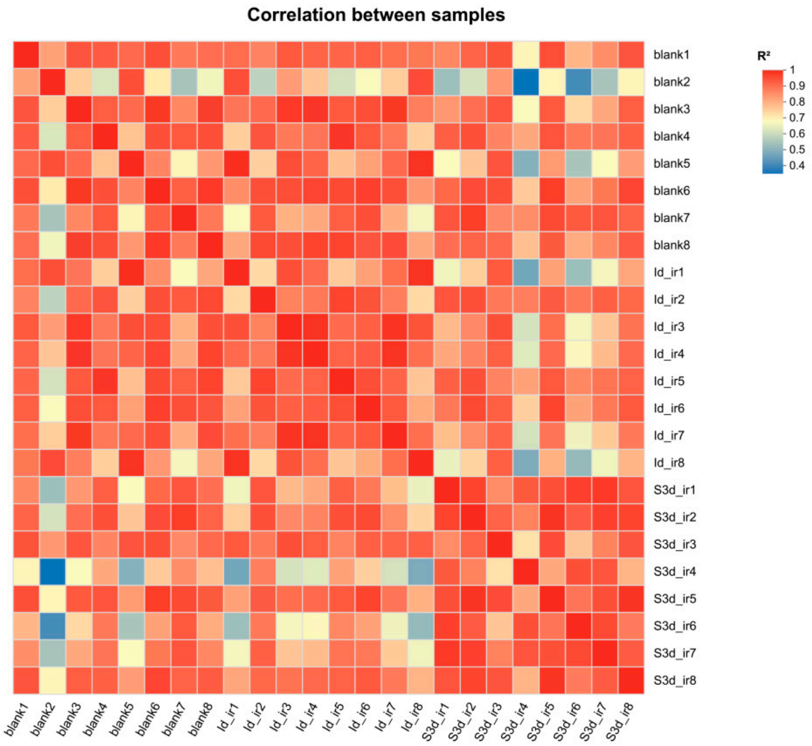

Figure S4. Sample Correlation Heatmap

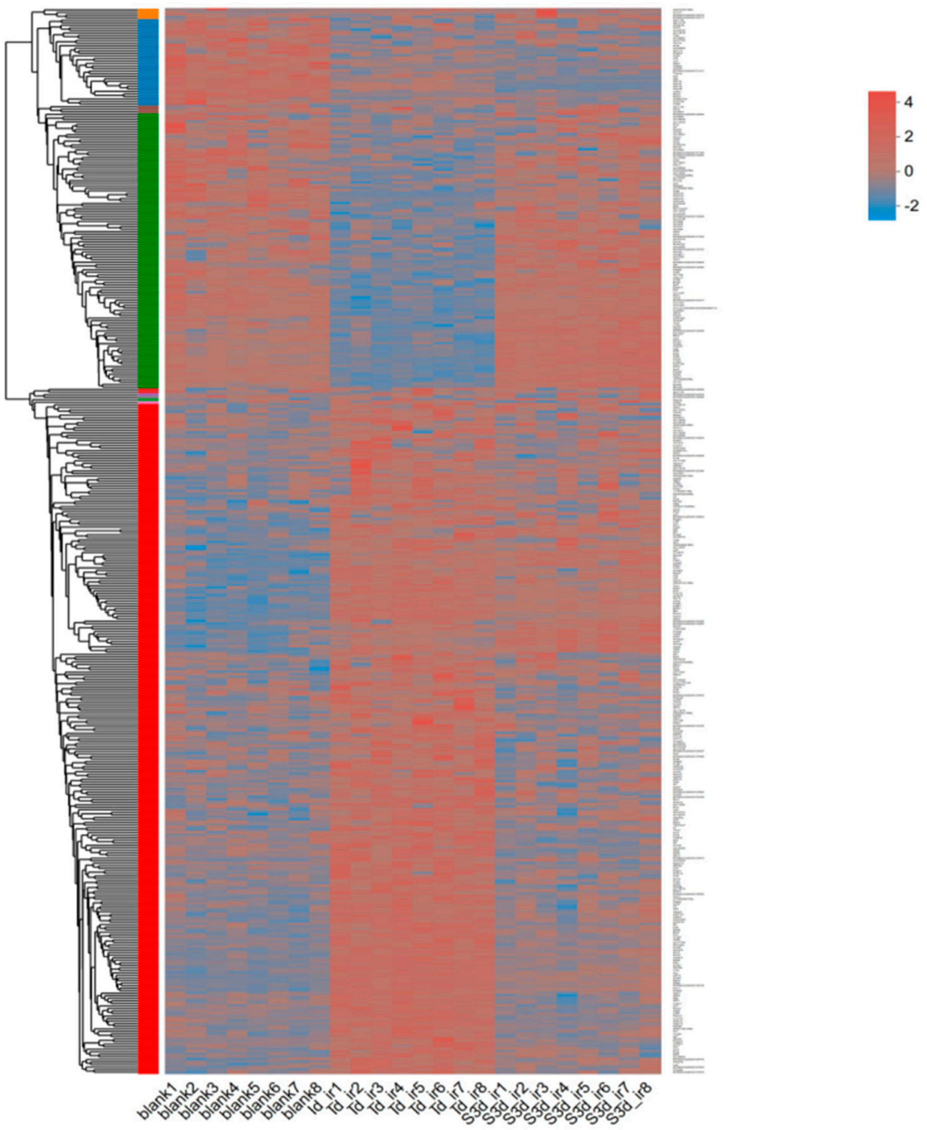

Figure S5. Clustering heatmap of differential gene expression in mouse small intestinal tissue among groups (n=8)

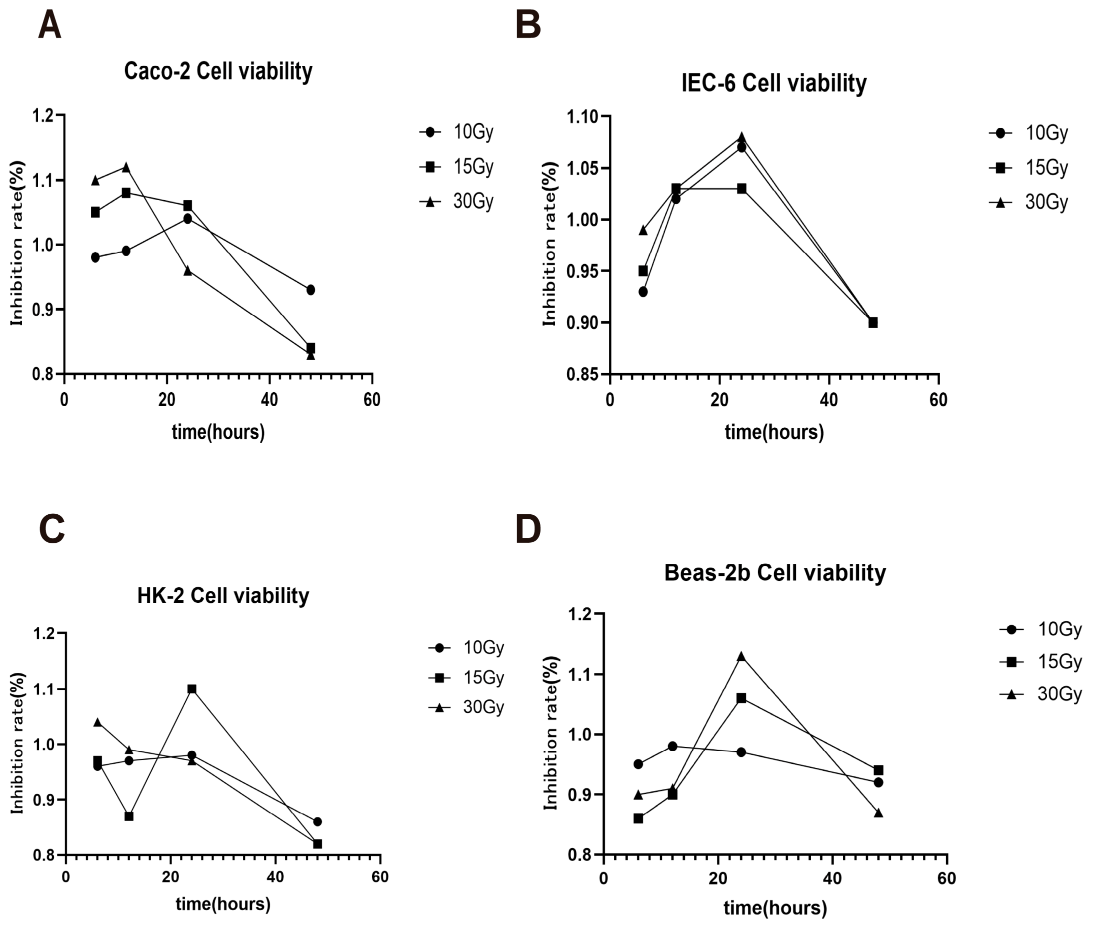

Figure S6. Four cell lines were subjected to MTT cell viability assays following exposure to various gamma-ray irradiation doses and at different post-irradiation time points. (n=6)
